# Breast cancer survivors` recollection of their quality of life: Identifying determinants of recall bias in a longitudinal population-based trial

**DOI:** 10.1371/journal.pone.0171519

**Published:** 2017-02-02

**Authors:** Patricia Lindberg, Petra Netter, Michael Koller, Brunhilde Steinger, Monika Klinkhammer-Schalke

**Affiliations:** 1 Tumor Center Regensburg, Institute of Quality Management and Health Services Research of the University of Regensburg, Regensburg, Germany; 2 Department of Psychology, University of Giessen, Giessen, Germany; 3 Michael Koller, Center for Clinical Trials, University Hospital Regensburg, Regensburg, Germany; Fu Jen Catholic University, TAIWAN

## Abstract

**Background:**

The recollections of survivors of breast cancer are an important source of information about the disease for their family, friends, and newly diagnosed patients. So far, little is known about these memories. This study investigated how accurately survivors of breast cancer remember their past quality of life (QoL) during the disease and if this memory is modified by women`s present QoL and negative affect.

**Material and methods:**

The longitudinal population-based study included 133 survivors of breast cancer (response rate 80%). Participants were asked for their present QoL and to recall their baseline QoL (EORTC QLQ-C30, QLQ-BR23) that had been assessed about seven years ago before discharge from hospital. The dependent variable was recall bias in ten QoL dimensions. Present QoL and negative affect (PANAS) were investigated as predictor variables.

**Results:**

Overall, baseline QoL was retrospectively underrated on seven out of ten scales whereas no significant overestimation was found. In multiple linear regression analyses, controlling for confounders, a stronger underrating of QoL was significantly predicted by a lower present QoL on nine out of ten scales and by higher negative affect on six scales.

**Conclusions:**

Survivors of breast cancer tend to underestimate their past QoL during the disease when asked about seven years later. Lower present QoL and higher negative affect contribute to this recall bias. This needs to be considered when interpreting retrospectively reported QoL data. Results are discussed in relation to theory of change or stability and mood congruency theory.

## Introduction

The number of long-term survivors of breast cancer has grown during the last years due to advances in early detection and therapy. [1] While attention has considerably focused on the investigation of long-term quality of life (QoL) [2], little is known how breast cancer survivors remember their past QoL during the time of the disease. But these recollections of breast cancer survivors can be highly relevant information for newly diagnosed patients and their families. It has been shown that healthy people tend to overestimate the emotional impact that a chronic disease like cancer will have on their lives (*disability paradox*) [3,4] and that the intensity and duration of the emotional reactions to such future events are overestimated (*affective forecasting*) [5]. This can affect therapy preferences and lead to refusal of essential therapies. [3] Affective forecasting can be improved when persons rely on information from others who have recently undergone the same experience. [6] Thus, the recollections of breast cancer survivors can contribute to shape the expectations of newly diagnosed women (e.g. support groups, internet).

When investigating memory for a subjective measure like QoL the criterion cannot be an exact agreement between remembered and actually reported data. Instead, it is far more relevant to investigate the occurrence and the direction of a systematic *recall bias*. The term recall bias is commonly used in epidemiologic studies describing the extent to which recall of the exposure to causal or preventive factors of a specific disease is impaired and whether the impairment is different for cases and controls. [7,8] In the present study recall bias is used to describe a systematic deviation of remembered and actually reported QoL. Earlier studies have demonstrated a recall bias for somatic symptoms showing a retrospective overestimation of symptom severity [9,10] and heterogeneous results with regard to prostate cancer patients’ assessments of QoL showing retrospective overestimation [11], underestimation [12], or no recall bias [13]. To the best of our knowledge, the recollection of QoL has not been examined in patients with breast cancer. For patients with non-cancer diseases it has been shown that QoL before a medical intervention is retrospectively underestimated so that patients perceive a larger subjective benefit of the intervention. [14–16]

Based on the *implicit theory of change or stability* [17] we hypothesized an association between present QoL and recall bias. According to this theory long-term memory for personal attributes like QoL encompasses two major steps: (1) the present status of the attribute serves as a benchmark because it is more available; (2) the past is constructed by characterizing it as different from or the same as the present, based on implicit theories of change or stability. [17] The difference between past and present should be overestimated when a person expects a change that is in fact very small or does not exist. [18] This assumption is strengthened when the time period between past and present is long. [19] Applying the theory to the recollection of QoL it should be expected that survivors of breast cancer remember their past QoL by using their present QoL as a benchmark. Past QoL is constructed based on an implicit theory of change, assuming that QoL has improved since receiving the diagnosis, because the disease has been successfully mastered. In fact, the changes in QoL, if any, should be only small because QoL declines with increasing age in most dimensions. [20] Therefore, a low present QoL should be related to an underestimation of past QoL.

Furthermore, negative affect, which implies negative emotionality and a negative self-concept [21], should contribute to recall bias. Studies have shown that persons scoring high on negative affect report a lower QoL and more symptom burden. [22–24] According to the theory of *mood congruency* [25] the present mood influences what kind of memory is retrieved (e.g. a sad mood triggers the recollection of negative memories). This has been demonstrated both for depressed patients [26,27] and for healthy subjects with experimentally induced depressed mood. [28] Likewise subjects with less negative affect report significantly more positive autobiographical memories (positivity bias). [29] Similar results have been found for neuroticism which comprises negative affect as one of its facets. [30,31] Based on this previous research it was assumed that survivors with higher negative affect remember a lower QoL, resulting in more underestimation of QoL.

In summary, this study proceeded with the hypothesis that survivors of breast cancer underestimate their past QoL during the disease and that a low present QoL and high negative affect contribute to this underestimation.

## Methods

### Participants

The study cohort consists of patients who took part in a randomized, controlled trial on the benefits of routine QoL diagnostics and therapy in breast cancer treatment. The trial included 200 female patients newly diagnosed with breast cancer who were recruited between 2004 and 2006 and surgically treated in one of five participating breast cancer centers in Bavaria, Germany (for more details see Klinkhammer-Schalke et al [32]). From August to December 2012 a follow-up of survivors of this sample was conducted (mean time since surgery 84 months; range 73–93 months). Of the 200 study participants 31 had died up to August 2012. Moreover, three women had to be excluded because of refusal during the former trial so that 166 survivors were eligible for follow-up. The term ‘survivor’ is here used as five year survival of the cancer diagnosis, a criterion commonly used in cancer statistics. [1,33]

### Design and procedure

A prospective, single group, cohort design was used combining data from a former trial [32] with new long-term follow-up data collected about seven years later. Ethical approval had been obtained from the local university ethics committee (University of Regensburg, 03/197) and patients had given their written informed consent.

Between 2004 and 2007 patients with breast cancer filled in a QoL questionnaire measuring their ***baseline*** QoL after surgery in the hospital 0 to 2 days before discharge. In 2012 a ***follow-up*** measurement was conducted about seven years after the baseline measurement. Survivors of this cohort were asked to recall their baseline QoL. For this purpose, all eligible women were sent a package of questionnaires supplemented by a cover letter explaining the purpose of the study and a stamped return envelope by post. An overall instruction indicated to fill in the questionnaires by oneself, in the predefined order, and in a quiet environment without interruptions. If women did not respond within six weeks they received a reminder. [34] There were no financial or other incentives. The questionnaire package also included three qualitative questions. Results of this qualitative analysis have been published elsewhere. [35]

### Measures

#### Quality of life

QoL was measured at baseline and at follow-up using the 30-item core module of the European Organization for Research and Treatment of Cancer Quality of Life Questionnaire (EORTC QLQ-C30) [36] and the 23-item module for patients with breast cancer (EORTC QLQ-BR23). [37] The questionnaire is a self-administration instrument measuring QoL on a 4-point Likert-scale (except the dimension ‘global QoL’ which is measured on a 7-point Likert-scale). Satisfying internal consistency, good retest-reliability as well as construct and clinical validity have been demonstrated. [36–38] Raw scores were uniformly transformed to scales ranging from 0 to 100 with higher scores indicating a better QoL. The questionnaire has a multidimensional structure including different multi- and single-item scales. Before the beginning of data collection ten multi-item scales were preselected for further data analysis because of their outstandingly high relevance for patients with breast cancer [32]: global QoL, physical functioning, role functioning, emotional functioning, cognitive functioning, social functioning, body image, pain, fatigue, arm symptoms. In this study internal consistencies for these ten scales ranged from .70 (cognitive functioning) to .91 (role functioning) for baseline QoL. For follow-up QoL nine of the ten scales had a good internal consistency (Cronbach`s alpha >.85) except the scale physical functioning (Cronbach`s alpha .62)

#### Recalled quality of life

At follow-up survivors were asked to remember their baseline QoL (i.e. the first time they filled in a questionnaire shortly before they had left hospital) (***recalled QoL***). This was measured by a 29-item short form of the EORTC QLQ-C30 plus QLQ-BR23 which included only those ten scales that had been preselected for data analysis (see above ‘Quality of life’), but the original length and order of these scales remained unaffected. The Likert-scale was supplemented by the category *no recollection* in order to prevent participants from guessing instead of recollecting their baseline QoL. Cronbach`s alphas of the short form ranged from .71 (physical functioning) to .91 (global QoL, fatigue, pain) in this study.

#### Negative affect

Negative affect was measured at follow-up using a German version of the Positive and Negative Affect Schedule (PANAS). [39,40] This 20-item questionnaire is composed of the two largely independent scales positive affect and negative affect each consisting of ten adjectives which are rated on a 5-point Likert scale (*very slightly or not at all* to *extremely*). Scores range from 0 to 50 with higher scores indicating higher negative or positive affect. The scale positive affect (i.e. amount of energy and social activities) was not used for analysis in this study because it has been shown that this scale is not associated with symptom reporting and QoL. [23] But data were still collected in order not to change the structure of the questionnaire. To measure negative affect as a trait variable the instruction *how you feel in general* was used. The internal consistency of the scale negative affect is satisfying for the English (.84) [39] and in the German version (.86). [40] In this study Cronbach`s alpha was .91.

#### Demographic and medical variables

Demographic and medical variables were collected at baseline and at follow-up. Demographic variables included age, relationship status, children, educational level, and employment status. At baseline the medical variables tumor stage, type of surgery, and therapies during the first postoperative year (chemotherapy, radiotherapy, endocrine therapy, anti-HER2 monoclonal antibody) had been collected. At the time of follow-up the variables cancer recurrence and time since surgery were assessed.

Questionnaires had been tested beforehand in a pilot survey and were presented in the following order: EORTC QLQ-C30, QLQ-BR23 (follow-up QoL), PANAS, demographic and medical variables, EORTC QLQ-C30, QLQ-BR23 short form (recalled QoL). Table 1 gives an overview of these measures including the time points when data were collected.

**Table 1:** Summary of measures including time points.

Measures	Baseline (2004–2006)	Follow-up (2012)
QoL (*EORTC QLQ-C30*, *QLQ-BR23*)	x	x
Recalled QoL (*EORTC QLQ-C30*, *QLQ-BR23*, short form)	-	x
Negative affect (*PANAS*)	-	x
Demographic and medical variables	x	x

Baseline = first questionnaire filled in 0 to 2 days before discharge from hospital; follow-up = about 7 years after baseline.

### Statistical analyses

All statistical analyses were conducted using SPSS version 19. The frequency of missing data was inspected for each item and a scale score was only computed if at least half of the items had been answered. [41] Casewise instead of listwise exclusion of missing data was used to preserve power and reduce risk of bias. [42] To analyze if participants differed as a function of their group membership in the former trial (intervention vs. control) their baseline QoL, their follow-up QoL, and demographic, and medical variables were compared using two-sample t-tests, Mann-Whitney *U*, or *Χ*^2^ tests depending on the scaling. Participants and non-respondents were compared regarding baseline QoL, demographic, and medical variables by using two-sample t-tests, Mann-Whitney *U*, or *Χ*^2^ tests depending on the scaling. To test for recall bias paired t-tests were performed to compare baseline QoL and recalled QoL. For each of the ten QoL scales a hierarchical multiple regression analyses was computed to test the association of recall bias with the predictors follow-up QoL and negative affect after controlling for covariates. Potential covariates (age, relationship status, educational level, employment status, time since surgery, type of surgery, tumor stage, cancer recurrence, chemotherapy, radiotherapy, endocrine therapy, anti-HER2 monoclonal antibody) were identified by calculating Pearson`s, point-biserial, or Spearman`s rank correlations with ***recall bias*** (difference baseline QoL–recalled QoL), follow-up QoL, and negative affect. Those potential covariates which significantly correlated with at least one of these three variables were entered into the regression model in step 1together with baseline QoL to control for floor/ ceiling effects on each QoL scale. In step 2 the two predictors of interest (follow-up QoL, negative affect) were added to assess how much additional variance they explained (*ΔR*^*2*^). Paired t-tests were used to compare follow-up QoL with baseline QoL and recalled QoL to test implicit theory of change or stability. A Bonferroni correction for multiple comparisons was applied where appropriate.

## Results

### Characteristics of participants

Of 166 eligible survivors 133 women participated in follow-up (response rate 80%). Demographic and medical characteristics of the participants are shown in Table 2.

**Table 2:** Demographic and medical characteristics of breast cancer survivors (N = 133).

Characteristics	N (%)	M (SD)
**Age**		64.2 (10.8)
**Educational level**		
Did not finish school	2 (2)	
Compulsory	62 (47)	
Advanced vocational	57 (43)	
University	10 (8)	
Other	2 (2)	
**Marital status**		
Married	101 (76)	
Unmarried	4 (3)	
Divorced	14 (11)	
Widowed	14 (11)	
**Children**		
Children	113 (85)	
No children	10 (8)	
Unknown	10 (8)	
**Employment status**		
Employed	49 (37)	
Retired/ not employed	80 (60)	
Unknown	4 (3)	
**Time since surgery (months)**		84.8 (5.6)
**Cancer stage at diagnosis**		
UICC 0	2 (2)	
UICC I	69 (52)	
UICC II (II a and b)	41 (31)	
UICC III (III a, b, c)	21 (16)	
**Surgical procedure**		
Breast conserving therapy	106 (80)	
Mastectomy	27 (20)	
**Treatment (first year after surgery)**		
Chemotherapy	94 (71)	
Radiotherapy	118 (89)	
Endocrine therapy	113 (85)	
Anti-HER2 monoclonal antibody	11 (8)	
**Recurrent cancer**	18 (14)	

Respondents (n = 133) did not differ from non-respondents (n = 33) in their baseline QoL or in any of the variables shown in Table 2. It is also noteworthy that there were no significant differences in any dimension of follow-up QoL between patients of the intervention and control group of the former trial [32]. Thus, the 133 patients were treated as a single cohort in all reported analyses.

### Comparison of baseline QoL and recalled QoL

Paired t-tests were used to investigate the difference between baseline QoL and recalled QoL. Results are shown in Table 3. In seven of the ten dimensions women recalled their baseline QoL as significantly worse than it actually was (physical, role, cognitive functioning, body image, fatigue, pain, arm symptoms). This indicates a significant retrospective underestimation of baseline QoL. In contrast, paired t-tests revealed no significant overestimation of baseline QoL on any scale (see Table 3).

**Table 3:** Results of paired t-Tests comparing baseline QoL and recalled QoL on ten scales of the EORTC QLQ-C30, QLQ-BR23.

	Baseline M (SD)	Recall M (SD)	t	df	95% CI	p
Global QoL	53.02 (22.97)	56.45 (20.27)	-1.47	123	[-8.05, 1.20]	.15
**Functioning:**						
Physical	78.92 (25.32)	66.87 (26.41)	**4.50**	124	[6.75, 17.36]	< .001
Role	71.85 (33.17)	41.46 (29.02)	**8.77**	118	[23.53, 37.26]	< .001
Emotional	49.89 (27.81)	45.16 (28.96)	1.68	128	[-0.84, 10.31]	.10
Cognitive	81.51 (26.06)	71.22 (32.25)	**3.50**	127	[4.47, 16.11]	< .001
Social	72.18 (30.36)	63.25 (34.14)	2.68	126	[2.34, 15.51]	< .01
Body image	83.07 (21.07)	66.93 (32.96)	**5.75**	124	[10.58, 21.69]	< .001
**Symptom:**						
Fatigue	66.49 (27.76)	40.99 (27.86)	**9.06**	123	[19.92, 31.06]	< .001
Pain	74.27 (28.04)	56.40 (30.73)	**5.21**	124	[11.08, 24.65]	< .001
Arm symptoms	67.49 (24.69)	52.62 (31.26)	**4.98**	120	[8.96, 20.80]	< .001

QoL = quality of life; baseline = first questionnaire filled in 0 to 2 days before discharge from hospital; recall = recall of baseline QoL about 7 years later; CI = confidence interval.

Significant t-scores after Bonferroni correction (p^Ɨ^ = p * 10) are presented in boldface.

### Follow-up QoL and negative affect as predictors of recall bias

Hierarchical regression analyses were performed to predict recall bias in the ten different QoL dimensions. Table 4 shows results of step 2 of the regression analysis (only significant covariates are presented). After controlling for baseline QoL and demographic and medical covariates the two predictor variables follow-up QoL and negative affect explained between 9% (physical functioning, *p* = .005 after Bonferroni correction) and 34% (body image, *p* < .001) of the additional variance. Follow-up QoL was a significant predictor of recall bias on nine out of ten scales (except physical functioning) with a lower follow-up QoL indicating more retrospective underestimation of baseline QoL. Higher negative affect significantly predicted underestimation of baseline QoL on six out of the ten scales (physical, role, emotional, cognitive, social functioning, pain). On the remaining three scales there was a trend in the same direction, but did not reach the conventional level of statistical significance. Of the controlled covariates a higher baseline QoL significantly predicted more underestimation on all ten scales. Furthermore, a younger age was significantly associated with underrating of baseline QoL on the scales global QoL, emotional, cognitive functioning, pain, and arm symptoms (see Table 4).

**Table 4:** Results of hierarchical regression analysis (step 2^‡^) predicting recall bias (baseline QoL—recalled QoL) on ten scales of the EORTC QLQ-C30, QLQ-BR23.

	Regression Coefficients B [95% CI]					
Model	Follow-up QoL	Negative affect	Baseline QoL	Age	Adj. *R*^*2*^	*ΔR*^*2*^
**Global QoL**	-0.37*** [-0.57, -0.17]	0.19 [-0.32, 0.70]	0.82*** [0.66, 0.97]	-0.66** [-1.12, -0.20]	.51	.10***
**Physical functioning**	-0.26 [-0.52, 0.00]	0.85** [0.25, 1.44]	0.75*** [0.57, 0.93]	-0.07 [-0.67, 0.53]	.39	.09***
**Role functioning**	-0.28* [-0.51, -0.06]	1.07** [0.33, 1.81]	0.84*** [0.69, 1.00]	-0.57 [-1.29, 0.15]	.52	.11***
**Emotional functioning**	-0.38** [-0.61, -0.16]	1.06** [0.33, 1.78]	0.75*** [0.59, 0.90]	-0.70** [-1.22, -0.18]	.57	.22***
**Cognitive functioning**	-0.54*** [-0.75, -0.33]	0.88** [0.22, 1.54]	0.75*** [0.57, 0.92]	-0.72* [-1.30, -0.13]	.46	.24***
**Social functioning**	-0.43*** [-0.65, -0.22]	1.17** [0.45, 1.88]	0.80*** [0.62, 1.00]	-0.62 [-1.29, 0.05]	.53	.18***
**Body image**	-0.68*** [-0.88, -0.49]	0.52 [-0.11, 1.45]	0.64*** [0.43, 0.85]	-	.53	.34***
**Fatigue**	-0.45*** [-0.65, -0.25]	0.30 [-0.33, 0.94]	0.81*** [0.64, 0.98]	-0.30 [-0.75, 0.14]	.46	.14***
**Pain**	-0.34*** [-0.53, -0.15]	1.02** [0.29, 1.75]	0.91*** [0.73, 1.10]	-0.77** [-1.28, -0.26]	.52	.14***
**Arm symptoms**	-0.61*** [-0.77, -0.44]	0.28 [-0.35, 0.90]	0.75*** [0.57, 0.93]	-0.74*** [-1.15, -0.32]	.55	.28***

^‡^Step 1: inclusion of covariates (not shown in the table); step 2: additional inclusion of follow-up QoL and negative affect; only significant covariates of step 2 (baseline QoL, age) are shown in the table; for a summary of controlled covariates see S1 Table.

QoL = quality of life; baseline = first questionnaire filled in 0 to 2 days before discharge from hospital; recalled = recollection of baseline QoL about 7 years later; follow-up = about 7 years after baseline.

B: p-value from t-test of individual parameter estimates.

ΔR^***2***^: p-value from F-test for significant change in R^2^ after including negative affect, follow-up QoL.

Significant change in R^2^ after Bonferroni correction (p^Ɨ^ = p * 10) is presented in boldface.

*p < .05;

**p < .01;

***p < .001.

### Comparison of follow-up QoL with baseline QoL and recalled QoL

**Fig 1:**
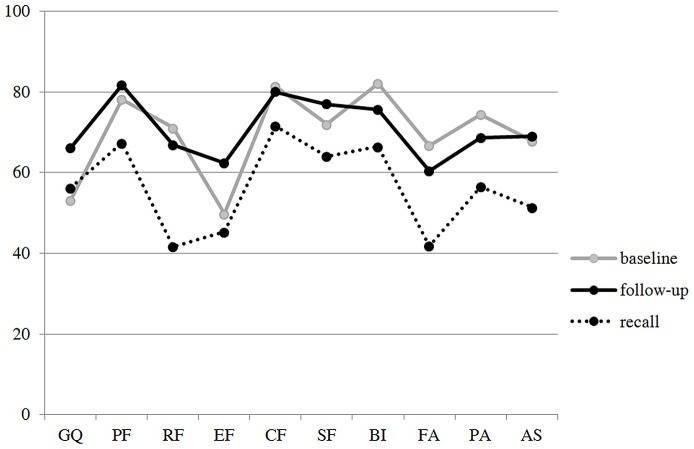
Baseline QoL, Follow-up QoL, and Recalled QoL on Ten Scales of the EORTC QLQ-C30, QLQ-BR23. Baseline = first questionnaire filled in 0 to 2 days before discharge from hospital; follow-up = about 7 years after baseline; recall = recollection of baseline QoL about 7 years later; GQ = global QoL; PF = physical functioning; RF = role functioning; EF = emotional functioning; CF = cognitive functioning; SF = social functioning; FA = fatigue; PA = pain; AS = arm symptoms; higher scores mean better QoL, reference values can be found in Hinz et al [20].

To test the theory of change or stability, predicting that baseline QoL is remembered by using follow-up QoL as a benchmark, we compared follow-up QoL with baseline QoL and recalled QoL (see Fig 1).

Bonferroni-adjusted paired t-tests showed that baseline QoL and follow-up QoL did not differ on most scales. Only global QoL (*t*(127) = 5.42, *p* < .001) and emotional functioning (*t*(129) = 4.42, *p* < .001) had significantly improved (see Table 5). These were also the dimensions in which survivors did not underestimate their baseline QoL (see Table 3). In the other eight dimensions there was no significant improvement or even a significant decline of follow-up QoL (body image: *t*(126) = 3.14, *p* < .01). Baseline QoL was significantly underestimated retrospectively in these dimensions (except social functioning after Bonferroni correction). In contrast, recalled QoL was significantly lower than follow-up QoL in all ten dimensions (see Table 5) indicating the use of follow-up QoL as a benchmark when recalling baseline QoL.

**Table 5:** Results of paired t-Tests comparing follow-up QoL with baseline QoL and with recalled QoL on ten scales of the EORTC QLQ-C30, QLQ-BR23.

	Follow-up -Baseline M (SD)	t	95% CI	Follow-up -Recall M (SD)	t	95% CI
Global QoL	12.96 (27.04)	5.42***	[8.23, 17.69]	10.33 (22.73)	5.08***	[6.31, 14.36]
**Functioning:**						
Physical	3.50 (25.68)	1.55	[-0.96, 7.96]	16.11 (26.58)	6.86***	[11.46, 20.76]
Role	-4.30 (38.95)	-1.23	[-11.22,2.62]	25.87 (34.42)	8.40***	[19.77, 31.96]
Emotional	12.54 (32.34)	4.42***	[6.93, 18.16]	16.99 (26.29)	7.37***	[12.43, 21.55]
Cognitive	-1.41 (31.06)	-0.52	[-6.80, 3.98]	8.79 (26.93)	3.71***	[4.09, 13.48]
Social	4.95 (35.70)	1.57	[-1.30, 11.19]	13.21 (29.33)	5.13***	[8.12, 18.29]
Body image	-7.83 (28.08)	-3.14**	[-12.76, 2.90]	8.31 (21.95)	4.30***	[4.49, 12.13]
**Symptom:**						
Fatigue	-6.38 (30.20)	-2.39*	[-11.66, -1.10]	18.60 (28.21)	7.49***	[13.69, 23.52]
Pain	-5.81 (36.24)	-1.82	[-12.13, 0.50]	12.14 (32.46)	4.25***	[6.49, 17.80]
Arm symptoms	1.09 (34.52)	0.36	[-4.97, 7.16]	16.22 (26.24)	6.91***	[11.58, 20.87]

Baseline = first questionnaire filled in 0 to 2 days before discharge from hospital; follow-up = about 7 years after baseline; recall = recollection of baseline QoL about 7 years later; CI = confidence interval.

*p < .05;

**p < .01;

***p < .001.

Significant t-scores after Bonferroni correction (p^Ɨ^ = p * 10) are presented in boldface.

## Discussion

To our knowledge this is the first study investigating the recollection of QoL during breast cancer in long-term survivors. Overall, survivors underestimated their baseline QoL after more than six years in most QoL dimensions. As predicted, a lower QoL at follow-up and higher negative affect contributed to this bias.

This underestimation of QoL is in line with previous studies investigating the recollection of QoL before a medical intervention in patients with non-cancer diseases. [14–16] In the current study recalled and actually reported QoL were significantly different in seven of the ten dimensions and these differences were also clinically meaningful with score point changes > 10 points. [43] Only in the dimension global QoL a weak, non-significant overestimation of QoL was found. An explanation may be, that this scale asks for a global résumé of one`s own well-being whereas all other scales measure specific symptoms and functions. This indicates that global QoL in the patient`s understanding implies more factors than the content represented by the symptom-specific subscales.

Our findings suggest that the disability paradox is not only prevalent in healthy persons imagining the emotional impact of a chronic illness on their lives [3], but also in the recollection of breast cancer survivors. Thus, survivors showed a tendency to overestimate the impact of breast cancer on their past subjective well-being. In the case of a cancer recurrence this overestimation of complaints may influence the acceptance of medical therapy and may lead to nocebo effects. When newly diagnosed patients with breast cancer rely on these negatively biased reports of survivors, affective forecasting [6] of patients may be also negatively biased by overestimating the intensity and duration of the emotional reactions to the disease. But it is possible that survivors report their illness experiences to newly diagnosed patients in a more positive way than in the present study to bolster them. This should be investigated in future studies with women expecting that their recollections serve as a source of information for patients with breast cancer.

Earlier studies [11–13] have discussed the phenomenon of *response shift* as an explanation for a distortion in cancer patients`recollection of their QoL. It describes a change of internal standards for evaluating QoL to adapt to the disease in order to maintain or improve QoL. [44] As a consequence, survivors should remember their baseline QoL as better than it actually was because internal standards have changed during the disease in order to maintain QoL. This is in contrast to our present finding of an underestimation of QoL that cannot be explained by response shift. Accordingly, recent studies have shown that response shift does not occur consistently and that the related effect sizes are only small. [45,46] An alternative explanation of our results might be that right after their surgery women were happy that surgery was over so that their baseline QoL was positively biased leading to the underestimation when recollecting QoL seven years later. However, it has been shown that QoL of patients with breast cancer is especially low after surgery [47] so that a positively biased QoL seems unlikely. The implicit theory of change or stability [17] gives a more suitable explanation for our findings predicting that survivors use their present QoL as a benchmark when remembering their past QoL based on the implicit theory that QoL has improved after the disease. In fact, only the two dimensions global QoL and emotional functioning had significantly improved since the baseline measure. These were also the dimensions in which no significant underestimation of baseline QoL could be found. In all other dimensions follow-up QoL was not better than baseline QoL or even had worsened (body image). An explanation for the absence of an improvement of baseline QoL may be a change of the reference group women use when evaluating baseline QoL and follow-up QoL by comparing themselves with other patients at baseline and with healthy women of the same age at follow-up. However, this seems to be unlikely when considering the conversational context in our study introducing survivors of breast cancer as the relevant reference group. Another explanation may be that QoL declines with increasing age. [20] Women in the present sample were six to eight years older at the time of follow-up. By underestimating their baseline QoL an improvement of follow-up QoL could be perceived anyhow. This was confirmed by our finding that recalled QoL was significantly lower than follow-up QoL on all ten scales. Likewise, follow-up QoL was found to be a significant predictor for the extent of underestimating baseline QoL in almost all dimensions (except physical functioning).

Furthermore, negative affect was found to be a significant predictor for recall bias in the six dimensions physical, role, emotional, cognitive, social functioning, and pain. The higher the negative affect the more the baseline QoL was underrated as predicted by mood congruency theory [25]. In the remaining four dimensions there was a non-significant trend in the same direction. These findings are in line with previous studies on neuroticism. [30,31] Thus, negative affect not only enhances a negative view on present QoL [22,24] but also influences the recollection of past QoL. Negative affect and follow-up QoL were identified as significant predictors for recall bias on five scales at the same time. This demonstrates that although both variables are related (see S2 Table) they still specifically contribute to account for independent proportion of variance of recall bias.

Of the controlled medical and demographic covariates only age was significantly associated with recall bias on the scales global QoL, emotional, cognitive functioning, pain, and arm symptoms, with younger women showing more underestimation of their baseline QoL. This can be explained by *positivity bias*, indicating that persons remember more positive than negative experiences with increasing age. [48]

The present findings have to be assessed with regard to the strengths and weaknesses of the study. To begin with, the study population was well-defined due to women`s participation in an earlier population-based trial. [32] Other strengths are the prospective long-term design and the high response rate more than six years after diagnosis. Furthermore, the study can claim high external validity [49] based on broad inclusion criteria. Several limitations should be considered. Self-administration instruments were used so social desirability may have influenced the results. Furthermore, it cannot be ruled out that women who refused participation had a very bad or very good follow-up QoL leading to a bias in the results. Fortunately, the rate of women who refused was low and those did not significantly differ from participants in their baseline QoL or in demographic or medical variables. The study also had no comparison group (e.g. healthy controls, survivors with other cancer diagnosis) so we do not know if the results are specific for survivors of breast cancer or can be generalized. Although missing data were no problem for most of the instruments used, there were missing values on the scale role functioning (EORTC QLQ-C30) of up to 11%. This is understandable because role functioning is difficult to evaluate for the time during hospital stay. It was decided not to exclude this scale to be consistent with the former trial [32] by analyzing the same ten scales in both studies.

In summary, the study demonstrated that survivors of breast cancer retrospectively tend to underestimate their postoperative QoL during the disease and that a lower present QoL and higher negative affect contribute to this bias. Such a negatively biased view on the disease may enhance survivors`fear of recurrence. Our findings are relevant for physicians who should address women`s worries and fears repeatedly during aftercare. In the case of a cancer recurrence the underestimation of QoL and by implication the overestimation of complaints may influence survivors`acceptance of medical therapy. This should be considered by clinicians during the medical encounter. Nevertheless, the individual experiences and memories of survivors of breast cancer are a valuable, indispensable source of information for newly diagnosed patients. In the light of the findings of this study it is important for patients to keep in mind that these subjective memories are prone to recall bias and should therefore not be accepted as facts. Instead, only those information and advices should be used which are helpful to the individual patient for better managing the disease.

## Supporting information

S1 Table Summary of controlled covariates^┼^ in hierarchical regression analysis. ^┼^covariates were included in the regression model (see Table 4) when they correlated significantly with recall bias (baseline QoL—recalled QoL), follow-up QoL, or negative affect; time since surgery, endocrine therapy, and marital status were no significant covariates; QoL = quality of life; baseline = first questionnaire filled in 0 to 2 days before discharge from hospital; follow-up = about 7 years after baseline; recalled = recollection of baseline QoL about 7 years later; GQ = global QoL; PF = physical functioning; RF = role functioning; EF = emotional functioning; CF = cognitive functioning; SF = social functioning; FA = fatigue; PA = pain; AS = arm symptoms.
(DOCX)

S2 Table Correlations between negative affect and follow-up QoL on ten scales of the EORTC QLQ-C30, QLQ-BR23. QoL = quality of life; follow-up = about 7 years after baseline measure; r = Pearson`s correlation; **p < .01; ***p < .001.
(DOCX)
