# Supplementary material for: Breast cancer survivors` recollection of their quality of life: Identifying determinants of recall bias in a longitudinal population-based trial
Source: PLoS One. 2017 Feb 2;12(2):e0171519. doi: 10.1371/journal.pone.0171519 (PMC5289621; doi:10.1371/journal.pone.0171519)
Supplement: S1 Table — ┼covariates were included in the regression model (see Table 4) when they correlated significantly with recall bias (baseline QoL—recalled QoL), follow-up QoL, or negative affect; time since surgery, endocrine therapy, and marital status were no significant covariates; QoL = quality of life; baseline = first questionnaire filled in 0 to 2 days before discharge from hospital; follow-up = about 7 years after baseline; recalled = recollection of baseline QoL about 7 years later; GQ = global QoL; PF = physical functioning; RF = role functioning; EF = emotional functioning; CF = cognitive functioning; SF = social functioning; FA = fatigue; PA = pain; AS = arm symptoms. (DOCX) [file pone.0171519.s001.docx]

| **Regression model (baseline QoL - recalled QoL)** | | | | | | | | | |  |
| --- | --- | --- | --- | --- | --- | --- | --- | --- | --- | --- |
| **Covariates** | **GL** | **PF** | **RF** | **EF** | **CF** | **SF** | **BI** | **FA** | **PA** | **AS** |
| **Baseline QoL** | x | x | x | x | x | x | x | x | x | x |
| **Age** | x | x | x | x | x | x | - | x | x | x |
| **Education** | x | x | x | x | x | x | x | x | x | x |
| **Employment status** | x | x | x | x | x | x | - | - | - | - |
| **Anti-HER2 monoclonal antibody** | x | x | x | x | x | x | x | x | x | x |
| **Tumor stage** | - | - | x | x | - | x | x | x | - | x |
| **Surgical Procedure** | - | - | - | - | - | x | x | - | - | - |
| **Chemotherapy** | - | - | - | - | - | x | - | - | - | - |
| **Radiotherapy** | - | - | - | - | - |  | x | - | - | - |
| **Recurrence** | - | - | x | x | - | x | x | - | x | x |
