# Supplementary material for: Breast cancer survivors` recollection of their quality of life: Identifying determinants of recall bias in a longitudinal population-based trial
Source: PLoS One. 2017 Feb 2;12(2):e0171519. doi: 10.1371/journal.pone.0171519 (PMC5289621; doi:10.1371/journal.pone.0171519)
Supplement: S2 Table — QoL = quality of life; follow-up = about 7 years after baseline measure; r = Pearson`s correlation; **p < .01; ***p < .001. (DOCX) [file pone.0171519.s002.docx]

|  | ***r*** |
| --- | --- |
| Global QoL | -.51*** |
| **Functional scales** |  |
| Physical | -.24** |
| Role | -.36*** |
| Emotional | -.72*** |
| Cognitive | -.45*** |
| Social | -.45*** |
| Body image | -.50*** |
| **Symptom scales** |  |
| Fatigue | -.43*** |
| Pain | -.42*** |
| Arm symptoms | -.42*** |
